# Sex specific differences in hepatic and plasma lipid profiles in healthy cats pre and post spaying and neutering: relationship with feline hepatic lipidosis

**DOI:** 10.1186/s12917-017-1152-y

**Published:** 2017-08-08

**Authors:** Chiara Valtolina, Arie B. Vaandrager, Robert P. Favier, Maidina Tuohetahuntila, Anne Kummeling, Isabelle Jeusette, Jan Rothuizen, Joris H. Robben

**Affiliations:** 1 Department of Clinical Sciences of Companion Animals, Faculty of Veterinary Medicine, Utrecht University, Yalelaan 108, 3584 CM Utrecht, The Netherlands; 2 Department of Biochemistry and Cell Biology, Faculty of Veterinary Medicine and Institute of Biomembranes, Utrecht University, Yalelaan 2, 3584 CM Utrecht, The Netherlands; 3 Research and Development, Affinity Petcare, Pl. Xavier Cugat, 2 Edificio D, 3ª, Planta, 08174 St. Cugat del Vallès, Barcelona, Spain

**Keywords:** Lipid dimorphism, Cats, Feline hepatic lipidosis, Sex hormones, Sphingomyelin

## Abstract

**Background:**

A link between lipid metabolism and disease has been recognized in cats. Since hepatic lipidosis is a frequent disorder in cats, the aim of the current study was to evaluate liver and plasma lipid dimorphism in healthy cats and the effects of gonadectomy on lipid profiling. From six female and six male cats plasma and liver lipid profiles before and after spaying/neutering were assessed and compared to five cats (three neutered male and two spayed female) diagnosed with hepatic lipidosis**.**

**Results:**

Intact female cats had a significantly lower level of plasma triacylglycerides (TAG) and a higher liver level of the long chain polyunsaturated fatty acid arachidonic acid (AA) compared to their neutered state. Both male and female cats with lipidosis had a higher liver, but not plasma TAG level and an increased level of plasma and liver sphingomyelin compared to the healthy cats.

**Conclusion:**

Although lipid dimorphism in healthy cats resembles that of other species, intact female cats show differences in metabolic configuration that could predispose them to develop hepatic lipidosis. The increased sphingomyelin levels in cats with lipidosis could suggest a potential role in the pathogenesis of hepatic lipidosis in cats.

## Background

In human medicine, the study and evaluation of lipid metabolites with the use of lipid profiling or “lipidomics” has become crucial in order to understand the role of lipids in the pathophysiology of lipid-related diseases [1, 2]. The role of lipids in the development and progression of numerous diseases in man, such as diabetes mellitus, obesity, non-alcoholic fatty liver disease (NAFLD), Alzheimer and arteriosclerosis is well recognised [3–5]. The recognition of sex specific genetic dimorphism in lipid metabolism has helped to identify risk factors and to develop more specific and targeted therapies [6, 7].

Pre-menopausal women are considered to possess a more favourable (less pro-coagulatory and less pro-atherogenic) plasma lipid profile compared to men and post-menopausal women, with lower plasma levels of triacylglyceride (TAG), total cholesterol (TC), and low density lipoprotein-cholesterol (LDL-C), and higher levels of high density lipoprotein-cholesterol (HDL-C) [8–11].

Parallels in lipid profiles between species have been demonstrated. The essential fatty acid (FA) profile is known to vary considerably between males and females in humans, mice and rats, indicating that sex lipid dimorphism occurs in different species [12–20]. When compared to their male counterpart, premenopausal women possess a higher percentage of the long chain polyunsaturated fatty acids (LCPUFA) arachidonic acid (AA 20:4 n-6) and docosahexaenoic acid (DHA, 22:6 n-3) in plasma and liver [20–22]. Also in rats, LCPUFA levels in plasma and liver are higher in intact female rats compared to males, while this difference disappears after gonadectomy [18, 20].

In man, mice and rats LCPUFA are synthesised from the essential FAs linoleic acid (LA 18:2 n-6) and α-linolenic acid (ALA, 18:3 n-3) by the sequential activities of desaturases (Δ5, Δ6) and elongases [23, 24]. Oestrogen has been shown to stimulate the FA desaturase activities [20, 25–29] and is also known to increase the activity of the hormone sensitive lipases, resulting in an increased level of circulating FA [28, 29]. Furthermore, oestrogen appears to be involved in lowering the plasma concentration of TAG and very low density lipoprotein (VLDL) triglycerides in premenopausal females [17, 30, 31].

In cats, the link between lipid metabolism and specific diseases has also been acknowledged. Conditions like Niemann-Pick in Siamese cats, obesity, diabetes mellitus, and hepatic lipidosis (HL) have been associated with an altered lipid metabolism in cats [32, 33]. Hepatic lipidosis is considered one of the most common liver diseases in cats and is characterised by severe accumulation of triglycerides in the liver, resulting in an impaired liver function [34, 35]. The pathogenesis of HL in cats is still not completely elucidated. The strict carnivorous diet and the unique protein and lipid metabolism of cats have been indicated as important predisposing factors in cats [36]. Cats, as other strict carnivores, possess a unique lipid metabolism, characterised by their limited ability, unlike other mammals, to synthesise AA from LA and eicosapentaenoic acid (EPA) and DHA from ALA due to limited desaturases Δ5 and Δ6 activities [37, 38].

It has been reported that feline HL has no breed, age or sex predisposition [34, 35], although in one study female cats have been reported to be more affected than their male counterparts [39]. This seems to be in contrast with what is known in other species, where females, especially the premenopausal woman and intact female rats and mice, seem to be more protected from lipid related disease than the postmenopausal/spayed female and their male counterparts. If however, as has been suggested before, female cats are more susceptible to the development of HL compared to males, the answer could also be found in a unique sex related lipid profiling.

The aim of the current study is to perform lipid profiling in intact and spayed/neutered healthy cats fed with a common commercial diet and to evaluate the effect of gonadectomy on the liver and plasma lipid profiles. Additionally, we compare lipid profiles from healthy cats to those in cats affected by HL in an attempt to elucidate possible predisposing factors in the development of HL.

## Methods

This study was approved by the Committee for the Ethical Care of Animals of the Utrecht University.

### Healthy cats

The healthy cats and the study design, diet, blood collection, hormone measurements, anaesthesia and surgical procedure, and liver biopsy taking have been described previously [40]. Plasma and pre and post spaying/neutering liver biopsies obtained in this study [40] were also used for analysis.

In summary, six intact females and six intact males client-owned cats admitted to the Department of Clinical Science of Companion Animals of the Faculty of Veterinary Medicine, Utrecht University (DCSCA) for spaying/neutering were considered for the study. Following confirmation of good health cats were neutered and spayed and a liver biopsy taken. The health status as well as blood parameters were evaluated again 4 weeks later during the second admission to the hospital and before the additional liver biopsy was performed. A study diet was fed to all healthy cats recruited in the study for a total of 8 weeks, starting four weeks before the first admission to the hospital and terminating after the second blood and liver tissue sampling 4 weeks after the spaying/neutering surgery.

For analysis and interpretation of the results, healthy cats were divided in four groups: intact males (before neutering, *n* = 6), intact females (before spaying, *n* = 6), spayed females (after spaying, *n* = 6), neutered males (after neutering, *n* = 6).

### Cats with hepatic lipidosis

Cats admitted from January 2013 to January 2014 to the DCSCA with suspected HL based on clinical symptoms (anorexia, vomiting and icterus) were considered for this study. In all cats ultrasonography showed diffuse echodensity of the liver compatible with HL. Hepatic lipidosis was confirmed based on cytological evaluation of fine needle aspirates of the liver. Only cats that were euthanized due to deterioration despite intense treatment and/or financial constraints of the owner were enrolled in the study. Owners’ consent was asked to be signed prior to consideration of the cats for the study.

In contrast to the healthy cats, cats with HL did not receive any special diet.

Plasma that remained from heparinised blood collected during hospitalisation for diagnostic and clinical monitoring was freshly stored at -70 °C in cryogenic vials (Corning Inc., Corning NY, USA) for lipidomics analysis.

In cats with HL two wedge liver biopsies were immediately collected post-mortem via laparotomy. As reported before (Valtolina et al.) one sample was fixated in 4% neutral-buffered formalin and the other liver biopsy was rinsed in normal saline (NaCl 0.9%), rapidly frozen in liquid nitrogen and then stored at −80 °C. The latter was used for the lipidomics analysis.

### Lipidomics analysis

For the lipidomic analyses the liver biopsies of the healthy cats pre- and post spaying/neutering and of the cats with HL were re-suspended in 350 μL of buffer A (250 mM sucrose 0.2 mM EDTA, 5 mM DTT, and 10 mM Tris/HCl pH 8.0) and homogenized by mechanical disruption with a pestle tightly fitting to an Eppendorf tube followed by sonication (10 s, amplitude 10 μm). Protein was determined in the homogenates by the BCA method Pierce® BCA protein assay kit (Thermo scientific, Rockford, IL, USA) with BSA as standard.

To 100 μl of homogenized liver biopsy or 100 μL of plasma, 200 pmol of tri-pentadecanoylglycerol (TAG 45:0) and 100 pmol of di-linolenoyl phosphatidylcholine (PC (18:3, 18:3)) were added as internal standards. Subsequently, lipids were extracted and separated in a neutral and phospholipid fraction by fractionation on a silica-G column as described [41]. In short, lipid extracts were dissolved in methanol/chloroform (1/9, *v*/v) and loaded on top of the silica column. Neutral lipids were eluted with two volumes acetone, dried under nitrogen gas and stored at −20 °C. In the neutral fraction, TAG species, cholesterol, cholesterol esters (CE), and retinyl esters (RE) were determined by HPLC-MS as described [41]. Typically, just before HPLC-MS analysis, the neutral lipid fraction was reconstituted in methanol/chloroform (1/1, *v*/v) and separated on a Lichrospher RP18-e column (5 μm, 250 × 4.6 mm; Merck, Darmstadt, Germany). A gradient was generated from acetonitrile to acetone/chloroform 85/15, *v*/v, at a constant flow rate of 1 ml/min. Mass spectrometry of lipids was performed using Atmospheric Pressure Chemical Ionization (APCI) on a Biosystems API-4000 Q-trap (MDS Sciex, Concord, Canada). The system was controlled by Analyst version 1.4.2 software (MDS Sciex, Concord, ON, Canada) and operated in positive ion mode. Full scan runs spectra were obtained from m/z 250-1100. Total TAG was determined by quantitating all ions between m/z 530 and 1050 with a retention time of TAG species and corrected for the presence of second and third isotope peaks. The percentage of TAG 56:6/7 was determined by quantitation of the ions with a m/z 905 and 907. TAG unsat/sat ratio was calculated after quantitiation of the ions with a m/z of 853 (TAG52:5), 879 (TAG54:6), 859 (TAG 52:2) and 885 (TAG54:3).

In the phospholipid fraction, sphingomyelin (SM) and PC species were determined as described [42]. The phospholipid fraction was dissolved in chloroform/methanol (1:1, *v*/v), and PC and SM molecular species were separated LiChrospher 100 RP18-e column (Merck), with a mobile phase of acetonitrile/methanol/triethylamine (25:24:1, *v*/v/v). Identification of molecular species was performed by on-line tandem mass spectrometry in the positive-ion mode on an API 4000 Q Trap mass spectrometer fitted with an electrospray ionization source (Sciex) by precursor scans of 184 m/z (choline head group).

Data analysis was performed using Analyst 1.4.2 software (MDS Sciex, Concord, ON, Canada) and calibration curves of all lipid classes were established under similar conditions as the samples.

### Statistical analysis

#### Healthy cats, pre versus post spaying/neutering

The outcome variables TAG, TAG unsat/sat, TAG56:6/7, cholesterol, cholesterol ester, total PC, PC species (% of total PC) and SM were respectively analysed using a linear mixed model with the cat identification as random effect to take repeated observations within the subject into account. “Time” (before/after spaying/neutering) and “gender” (male/female) and the interaction between time and gender were used as explanatory variables. The Akaike Information Criterion (AIC) was used to select the best model. Residuals plots were used to assess the validity of the model by visual inspection of the QQ-plot of residuals for normality and scatterplots of residuals versus predicted values for constant variance.

Some outcome variables (liver TAG sat/unsat; plasma PC 34:1) were log transformed to meet the model assumptions normality and constant variance. In this model we also could take the correlation between observations into account.

If data were not normally distributed, neither after log transformation (liver TAG 56:6/7; liver PC 38:6; plasma TAG sat/unsat), a nonparametric Kruskall and Wallis test was applied to assess the difference in means between groups of combined gender and time (preMale, preFemale, postMale en postFemale respectively) and a non-parametric Wilcoxon test was applied to assess the difference within genders between both times. Unfortunately this model was not able to take the correlation between observations into account.

In order to identify factors involved in the variation in liver TAG levels in the healthy cats a Pearson correlation analysis was performed.

#### Healthy cats versus cats with hepatic lipidosis

Comparison between the mean of the respective outcomes of the healthy cats after spaying/neutering and cats with HL were calculated with the independent Student’s *T*-test.

*P*-value <0.05 was used to assess statistical significance. Results are expressed as mean and standard deviation (SD), if not stated otherwise.

Data analysis was performed using IBM SPSS 22 statistic software (IBM Corporation Armonk, NY, USA).

## Results

### Animal’s characterization

A description of the healthy cats has been previously reported [40]. In summary, six healthy females (20.5 (6.0 – 84.0 months); 3.0 (2.6 – 3.9) kg; (median (range)) and six healthy males (7.0 (6.0 – 9.0) months; 4.0 (3.5 – 4.5) kg; (median (range)) were enrolled in this study Results of the CBC, biochemistry and coagulation profile of all 12 cats were within reference intervals. Both oestrogen in the female group (*P* = 0.041) and testosterone in the male group (*P* = 0.001) dropped significantly confirming successful gonadectomy [40].

Histological evaluation of the liver biopsies performed in the healthy cats (pre and post spaying/neutering) at 4 weeks and at 8 weeks after the beginning of administration of the diet, revealed no histological changes compatible with HL or any other pathological change in any of the healthy cats [40].

Two spayed females (50.0 and 52.0 months; 3.5 kg and 4.5 kg, respectively), and three neutered males (38.0, 40.0, and 48.0 months; 3.0, 4.0, and 4.3 kg, respectively) with HL were enrolled in this study. During a period of one month to three days prior to admittance cats showed clinical signs consisting of, but not limited to, lethargy, anorexia, vomiting, and weight loss. Diagnosis of HL during life was confirmed with cytological examination of liver fine needle aspirates performed by a diplomate in European College of Veterinary Clinical Pathology.

### Lipid profiles

#### Healthy cats, pre- and post-spaying/neutering

Plasma level of TAG was almost one-and-a-half-fold lower (*P* = 0.017) in intact female cats compared to intact male cats (Table 1). There was also a significant difference of the plasma level of TAG between males pre and post neutering, where neutered male cats had higher plasma levels of TAG compared to the intact males (*P* = 0.033).

**Table 1:** Effect of sex hormones and spaying/neutering on liver and plasma lipids of healthy cats (means ± SD)

	Liver				Plasma			
	Group 1	Group 2	Group 3	Group 4	Group 1	Group 2	Group 3	Group 4
	Male intact (*n* = 6)	Male neutered (*n* = 6)	Female intact (*n* = 6)	Female spayed (*n* = 6)	Male intact (*n* = 6)	Male neutered (*n* = 6)	Female intact (*n* = 6)	Female spayed (*n* = 6)
Total TAG^1^								
(nmol/mg prot)	51 ± 35	58 ± 45	99 ± 70	78 ± 52				
(mmol/l)					0.22 ± 0.05^abc^	0.30 ± 0.10 ^abc^	0.15 ± 0.03 ^ac^	0.14 ± 0.05 ^ac^
TAG 56:6/7^2^ (%)	0.6 ± 0.2	0.8 ± 0.4	0.7 ± 0.1 ^b^	0.5 ± 0.1^b^	0.7 ± 0.3	0.8 ± 0.3	0.8 ± 0.2	0.7 ± 0.2
TAG unsat/sat^3^	1.0 ± 0.3	1.1 ± 0.4	1.1 ± 0.4	0.8 ± 0.3	1.8 ± 0.4	1.8 ± 0.2	2.0 ± 0.6	1.8 ± 0.4
Cholesterol (rel. Units)	13.1 ± 1.7	14.2 ± 1.3	15.3 ± 1.6	13.6 ± 2.0	8.9 ± 0.7	9.9 ± 1.2	9.3 ± 1.1	9.0 ± 0.6
Cholesterol ester (rel. Units)	7.8 ± 2.9	7.6 ± 2.6	10.3 ± 4.6	9.1 ± 3.0	61 ± 8	62 ± 10	56 ± 6	59 ± 3
Total PC^4^								
(nmol/mg prot)	24.2 ± 3.8	24.1 ± 3.2	27.5 ± 3.1	24.1 ± 2.3				
(mmol/l)					0.16 ± 0.02	0.17 ± 0.03	0.15 ± 0.03	0.15 ± 0.03
SM^5^ 16:0 (% of PC)	3.8 ± 0.8	3.8 ± 0.5	3.4 ± 0.9	3.9 ± 0.8	7.4 ± 0.7	6.9 ± 0.5	7.4 ± 0.7	7.5 ± 1.1

1. TAG = triacylglycerol

2. TAG 56:6/7 = TAG containing relative long unsatured acyl chains

3. TAG unsat/sat = the ratio between TAG with 5 or 6 double bonds versus 2 or 3 double bonds

4. PC = phosphatidylcholine

5. SM = sphingomyelin (SM)

a *P* < 0.05 male vs female (gender)

b = *P* < 0.05 intact vs spayed/neutered (time)

c = *P* < 0.05 interaction between gender and time

In contrast, intact female cats had two-fold higher TAG levels in the liver compared to intact males (Table 1). Due to a large variation, this difference did not reach statistical significance (*P* = 0.17). After castration the TAG liver levels in the female cats dropped, but were still higher (but not significant), compared to the TAG levels in castrated males (Table 1).

The Pearson correlation analysis demonstrated the highest ranked correlations for liver TAG levels with PC 36:4 in both the liver and plasma (*r* = +0.62; *P* = 0.001 for liver PC36:4, and *r* = +0.540; *P* = 0.006 for plasma PC36:4). A significant positive correlation (*r*) of liver TAG levels with liver cholesterol esters was also observed (*r* = +0.59; *P* = 0.003), but no significant correlation was found between plasma and liver TAG levels (*r* = − 0.19; *P* = 0.39).

**Fig. 1:**
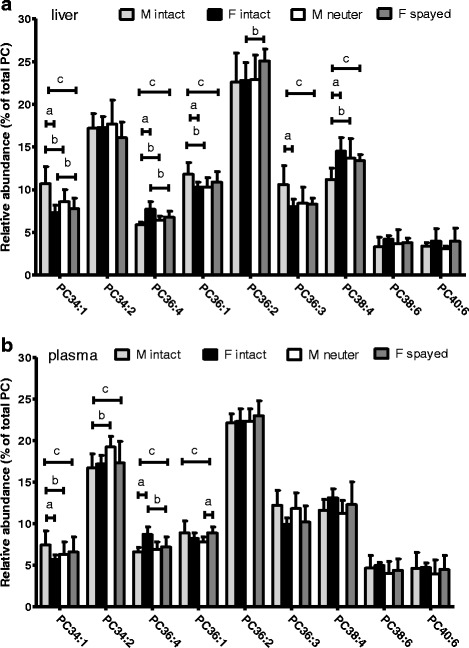
Effect of sex (gender), spaying/neutering (time) and their interaction (gender x time) on species distribution of phosphatidylcholine (means ± SD). The major phosphatidylcholine (PC) species were determined in liver biopsies (**a**) and plasma (**b**). PC36:4 and PC38:4 contain arachidonic acid (20:4). PC38:6 and PC40:6 contain docosahexaenoic acid (22:6). a = *P* < 0.05 male vs female (gender), b = *P* < 0.05 intact vs spayed/neutered (time), c = *P* < 0.05 interaction between gender and time

**Fig. 2:**
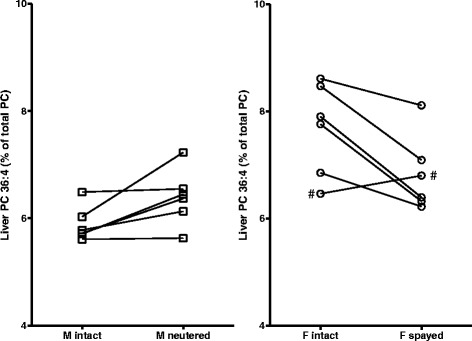
Effect of sex and castration on palmitoyl-arachidonoyl-phosphatidylcholine (PC36:4). The phosphatidylcholine (16:0, 20:4) species (PC36:4) were determined in liver biopsies from 6 cats per group. Significant differences were present in liver PC 36:4 between male and female cats (gender) and between male cats pre and post neutering and female cats pre and post spaying. Notice how in the male cats, neutering increases the PC 36:4 liver values. In female cats, spaying decreases the PC 36:4 liver values. # intact cat had low estrogen level

The total levels of PC in plasma and liver did not differ significantly between male and female cats, both before and after spaying /neutering (Table 1). The overall species profile of PC in plasma was similar to that in liver tissue (Fig. 1). The most abundant PC species were PC34:2 and PC36:2, both predominantly containing linoleic acid (18:2) in combination with palmitic acid (16:0) (PC34:2) or stearic acid (18:0) (PC36:2). There were differences in PC species profiles between the male and female cats before and after spaying/neutering (Fig. 1). The most significant differences in PC species between female and male cats were with those with an AA (20:4). The sum of the percentage of PC containing LCPUFA AA, PC36:4 and PC38:4, in intact female cats were significantly (*P* < 0.01 and *P* < 0.002, respectively) higher in both plasma (21.8 ± 1.6%) and liver (22.2 ± 2.3%), compared to male (plasma: 18.2 ± 1.7%; liver: 17.1 ± 1.4%) (Figs. 1 and 2). These percentages were also higher in both plasma and liver of intact female cats were compared to spayed female cats (plasma:19.5 ± 2.7%; 20.2 ± 1.2% liver), however the differences were not statistically significant (*P* < 0.08 and *P* < 0.1 respectively) (Figs. 1 and 2). The lower levels of AA-containing PC species in intact male cats coincided with higher levels of PC34:1 and PC36:3, predominantly containing non-PUFAs (16:0, 18:1, and 18:2). Phosphatidylcholine species with another abundant LCPUFA, i.e. DHA (22:6 n-3), PC38:6 and PC40:6, did not differ between intact males and females and castrated males and females. After spaying/neutering, the levels of PC36:4 dropped significantly in female cats and increased significantly in male cats (Figs. 1 and 2), resulting in similar levels of the AA-containing PCs in male and female cats after spaying/neutering.

#### Healthy cats versus cats with hepatic lipidosis

The cats with HL had the same level of plasma TAG as the healthy spayed/neutered cats, but an almost ten-fold higher level of TAG in the liver (*P* = 0.004) (Table 2). The levels of AA-containing PC species (36:4; 38:4), which were found to correlate positively with the liver TAG levels in healthy cats, were not higher in the HL cats and were even lower for PC 38:4 in the liver (see Table 2). Sphingomyelin (SM) (16:0) as a percentage of total PC, was significantly higher (two-fold) both in plasma and in liver when compared to the healthy cats.

**Table 2:** Comparison of liver and plasma lipids between healthy and cats with hepatic lipidosis (HL)

	Liver		Plasma	
	Cats spayed/neutered	HL cats	Cats spayed/neutered	HL cats
	(*n* = 12)	(*n* = 5)	(*n* = 12)	(*n* = 5)
Total TAG^1^				
(nmol/mg prot)	68 ± 48	584 ± 277^a^		
(mmol/l)			0.22 ± 0.11	0.40 ± 0.25
%TAG 56:6/7^2^	0.7 ± 0.3	1.0 ± 0.3^a^	0.8 ± 0.3	1.8 ± 1.4
TAG unsat/sat^3^	1.0 ± 0.4	1.0 ± 0.7	1.8 ± 0.3	1.0 ± 0.7
Total PC^4^				
(nmol/mg prot)	24.1 ± 2.7	10.2 ± 5.0 ^a^		
(mmol/l)			0.16 ± 0.03	0.16 ± 0.04
% PC^4^ 34:2	16.9 ± 2.4	20.7 ± 4.0	18.3 ± 2.2	19.3 ± 1.9
% PC 34:1	8.2 ± 1.3	12.5 ± 4.3	6.4 ± 1.6	12.8 ± 7.0
% PC 36:4	6.6 ± 0.6	5.3 ± 1.5	7.1 ± 1.0	5.8 ± 2.0
% PC 36:3	8.4 ± 1.4	6.4 ± 2.5	11.0 ± 2.0	8.4 ± 2.3^a^
% PC 36:2	24.0 ± 2.5	24.0 ± 6.2	22.6 ± 1.6	22.8 ± 3.0
% PC 36:1	10.6 ± 1.1	11.6 ± 3.6	8.4 ± 0.9	10.2 ± 2.7
% PC 38:4	13.6 ± 1.7	8.8 ± 3.9 ^a^	11.8 ± 2.2	10.0 ± 3.2
% PC 38:6	3.8 ± 1.1	3.7 ± 1.7	4.2 ± 1.4	4.0 ± 1.5
% PC 40:6	4.0 ± 1.6	3.7 ± 2.0	4.2 ± 1.6	3.7 ± 2.0
SM 16:0^5^ (% of PC)	3.8 ± 0.6	8.0 ± 3.5 ^a^	7.2 ± 0.9	14.2 ± 6.4^a^

1. TAG = triacylglycerol

2. TAG 56:6/7 = TAG containing relative long unsatured acyl chains

3. TAG unsat/sat = the ratio between TAG with 5 or 6 double bonds versus 2 or 3 double bonds

4. PC = phosphatidylcholine

5. SM = sphingomyelin (SM)

a = *P* < 0.005 HL vs healthy cats

## Discussion

Butterwick et al. has reported no important differences in plasma lipid and lipoprotein concentrations between sexually intact females and males or between sexually intact and castrated males have been demonstrated [43]. However, as sexual dimorphism in the lipid metabolism has been demonstrated so clearly in different species, we wanted to explore this further in cats. In a recent study we were unable to demonstrate significant differences in liver PC levels between male and female cats pre- and post- spaying/neutering and a lack of evident influence of sex hormones on the PC synthesis by the phosphatidylethanolamine N-methyltransferase (PEMT) pathway [40]. However, other lipid factors might be involved if a potential sexual dimorphism in cats exists. To evaluate if a sexual dimorphism could still be established and if differences could be related to HL, we evaluated in this study the lipid profile in cats in more detail.

Intact female cats have significantly lower TAG levels in plasma compared to their male counterparts, similarly to premenopausal women, intact female rats and mice [12–15, 18, 19]. However, this difference was not reversed by spaying but rather enhanced due to a rise in the plasma TAG level in male cats after neutering.

Although not significant possibly due to large variation, the liver TAG levels were lower in male cats compared to female cats. The significantly higher plasma TAG levels in male cats may be related to a higher rate of apolipoprotein B and VLDL synthesis by hepatocytes in male cats [30, 31], as the TAG levels in plasma most likely reflect the VLDL fraction [44]. Also a reduced plasma clearance of VLDL triglyceride in male cats compared to their female counterparts cannot be excluded. Interestingly, in premenopausal women a lower concentration of VLDL-TAG and LDL particles compared to men seems to be associated with accelerated (rather than reduced) VLDL-TAG and LDL production and increased plasma clearance [30, 31, 45–48]. Our observations warrant further studies into gender specific VLDL secretion/breakdown in cats.

The results of the lipidomic analysis in the cats with HL in this study confirm findings in previous studies analysing lipids in liver and in plasma [49, 50]. We also observed that cats with HL have significantly higher TAG levels in their liver. However, in our study cats with HL had no significantly different plasma TAG levels compared to healthy cats, whereas in other studies high plasma TAG levels in cats with HL have been described [39, 49, 50]. The secretion of VLDL in HL may prevent lipid accumulation in the liver by exporting the surplus TAG and it can be stimulated by higher levels of TAG in the liver of cats with lipidosis [44]. However, the results in the cats with HL suggest there may be a maximum capacity of the liver in cats to secrete VLDL, so that no further increase in plasma VLDL is observed in association with high liver TAG levels [51].

Intact female cats have a higher content of LCPUFA AA (20:4 n-6) in PC in both plasma and liver tissue, when compared to spayed female and the male counterparts. This finding is similar to what has been reported in premenopausal women, intact female rats and mice [12–15, 18, 19]. However, in contrast to what has been reported in these species, PC levels containing LCPUFA DHA (22:6 n-3) were not elevated in intact female cats [18, 21, 52, 53]. Despite this difference, there seems an influence of sex hormones on the profile of PC species in cats likely as the differences between the sexes disappeared after spaying/neutering.

In humans, the increased LCPUFA levels appear to be related to an oestrogen effect on FA desaturase activity via increased expression of Δ5 and Δ6 desaturase [18, 25, 26]. Although cats were believed to have low Δ5 and Δ6 desaturase activity [38, 54], in a more recent study cats were able to synthetize AA, but not DHA, from the substrate γ-linolenic acid (18:3 n − 6) via Δ5 desaturase, bypassing the Δ6 desaturase step [37]. This is supported by findings in this study as the primary increase in AA but not DHA levels in the liver of intact female cats compared to the male and spayed female counterparts might also suggest a possible role of Δ5 desaturases.

In humans and mice the higher LCPUFA levels may protect against the development of HL via the so-called “fuel partitioning action” of LCPUFA [55–57]. Long chain polyunsaturated fatty acids favour FA oxidation over TAG storage and they direct glucose away from FA synthesis by facilitating glycogen synthesis. However, the n-3 LCPUFA species (i.e. DHA), rather than the n-6 LCPUFA (i.e. AA), are mainly responsible for these effects via the activation of the peroxisome proliferating receptors (PPARs) in liver and adipose tissue [57–60]. The low liver levels of n-3 LCPUFA in the intact female cats may suggest less resistance to HL compared to females of other species.

In contrast to what it would be expected, in the cats with HL in this study, we did not find direct evidence for an increase in the n-6/n-3 ratio as indeed, the AA-containing PC 38:4 in cats with HL was even significantly lower compared to healthy cats and there was no difference in n-3 LCPUFA between HL and healthy cats. In cats with HL a lower percentages of the LCPUFA (i.e. AA) in the liver have been demonstrated before [49, 50].

Sphingomyelin, a membrane lipid primarily present in the membranous myelin sheath that surrounds nerve cell axons, and its relation in the development of steatosis has been observed in different animal models of obesity and fatty liver [61, 62]. The increased synthesis of SM in livers affected by NAFLD promotes hepatic insulin resistance, hepatocyte apoptosis, an increased release of inflammatory mediators, and ceramide accumulation in peripheral tissues [63]. The increased SM concentrations, both in liver and in plasma, in our cats with HL warrants further study in order to understand the potential role of SM in feline HL.

## Conclusions

Although some differences are found, the sexual dimorphism in the hepatic and plasma lipid profile of healthy cats resembles observations in other species with lower plasma TAG levels and increased plasma and liver AA in intact females compared to males. However, the higher plasma and liver levels of AA but not DHA could predispose intact female cats for HL. Also spaying of female cats may not increase the risk for HL.

In contrast to previous studies, the higher liver but not plasma TAG levels in cats with HL compared to healthy cats could suggest a maximum capacity for liver excretion of VLDL. The increased liver and plasma SM concentration in cats with HL compared to healthy cats could indicate a novel mechanism in the development of HL in cats.

## Abbreviations

AA: Arachidonic acid
DHA: Docosahexaenoic acid
FA: Fatty acid
HDL: High density lipoprotein
HL: Hepatic lipidosis
LA: Linoleic acid
LCPUFA: Long chain polyunsaturated fatty acid
LDL: Low density lipoprotein
NAFLD: Non-alcoholic fatty liver disease
PC: Phosphatidylcholine
PEMT: Phosphatidylethanolamine N-methyltransferase
SM: Sphingomyelin
TAG: Triglyceride
TC: Total cholesterol
VLDL: Very low density lipoprotein
Δ: Desaturase
